# Activity-Based Profiling of Papain-like Cysteine Proteases in Different Plant Organs During Barley Development

**DOI:** 10.3390/plants15101523

**Published:** 2026-05-16

**Authors:** Igor A. Schepetkin, Andreas M. Fischer

**Affiliations:** Department of Plant Sciences and Plant Pathology, Montana State University, Bozeman, MT 59717, USA

**Keywords:** activity-based protein profiling, barley development, germinated seed, *Hordeum vulgare* L., papain-like cysteine protease, protein purification, senescence, tandem mass spectrometry

## Abstract

Papain-like cysteine proteases (PLCPs) are vital enzymes involved in plant development, acting as key regulators of processes such as seed germination, nutrient mobilization, senescence, and programmed cell death. In the present study, we analyzed active PLCPs in various barley organs, including roots, leaves, stems, and seeds at different stages of plant development. Protein extracts obtained from barley samples (4-day-old seedlings; plants at 2, 4, 7, and 11 weeks after sowing; developing seeds from 11-week-old plants; and mature dry seeds) were subjected to anion-exchange chromatography. Fractions containing active PLCPs were pooled, biotinylated using the DCG-04 probe, affinity-purified using streptavidin-agarose, and subsequently analyzed via SDS-PAGE. Bands corresponding to biotinylated PLCPs (detected using streptavidin-peroxidase and a chemiluminescent substrate) were excised from the gel and analyzed by tandem mass spectrometry, enabling the identification of up to 23 distinct PLCPs belonging to nine known PLCP subfamilies. Among the identified PLCPs, HvPap-6 from the L-like D subfamily proved to be the most abundant across all barley samples. In seedlings, B-like and L-like D proteases constituted the largest proportion of all PLCP classes, and their levels continued to increase as the plants developed. Although the relative abundance of L-like B and L-like C proteases was high in seedlings, their levels declined in the roots and leaves of developing plants, as three PLCPs from the L-like B subfamily were identified only during the seedling stage. These results suggest that L-like B and L-like C proteases play an important role in seed germination and seedling development. Organ-specific expression was also observed for certain PLCPs: HvPap-26 from the L-Like C subfamily was identified only in the shoots and roots of seedlings; four PLCPs of the L-like E subfamily were detected solely in the roots, whereas two other proteases from this subfamily were identified exclusively in the leaves and shoots under our experimental conditions. Thus, our results suggest that certain active PLCPs are organ-specific, and that the relative importance of identified PLCPs varies within these organs during plant development.

## 1. Introduction

Papain-like cysteine proteases (PLCPs; clan CA, family C1A in the MEROPS protease database [1]) constitute one of the most abundant groups of proteases involved in plant development, regulating critical processes such as leaf senescence, seed germination, and programmed cell death [2]. They facilitate nutrient mobilization, xylem development, and stress responses, and act as central nodes in plant immunity, protecting plants from pathogens [3,4,5,6]. PLCPs exhibit diverse cellular and subcellular localizations, including the vacuole [7], apoplast [8], and chloroplast [9]. These proteases are characterized by a globular structure composed of two distinct domains, or lobes, separated by an active-site cleft. The N-terminal domain contains a set of α-helices while the C-terminal domain is rich in β-strands, usually paired with α-helices in a specific folding pattern. The active site, which contains a catalytic triad (Cys, His, and Asn/Asp), is located at the interface of these two domains, forming a V-shaped cleft [10]. Some PLCPs carry a signal peptide for vacuolar targeting or retention in the endoplasmic reticulum [11,12]. Based on similarity to cathepsins, all PLCPs can be classified as cathepsin L-, B-, H-, or F-like proteases, and L-like proteases are further divided into five groups (L-like A–E) [13].

Understanding the functional roles of PLCPs during plant development and senescence provides foundational knowledge for the improvement of crop traits, including seed/grain protein content and nitrogen use efficiency [14,15,16]. In barley, PLCPs have been classified as HvPap-1 to HvPap-42 [17]. Significant upregulation of several *HvPap* genes has been reported during barley leaf senescence and seed germination [17,18,19,20,21,22]. PLCPs are of particular importance for the degradation and mobilization of seed storage proteins [3,23]. Early studies of the barley grain germination process have identified several PLCPs [24,25]. Among these, EP-A, EP-B1, and EP-B2 were characterized [26,27,28]; according to current classification, they belong to the L-like B subfamily and correspond to HvPap-9 (EP-A) and HvPap-10/HvPap-11 (EP-B1/EP-B2). These proteases are secreted from the aleurone layer and scutellar epithelium into the endosperm, where they cleave the storage proteins, providing the seedlings with amino acids [28]. Subsequent studies confirmed the expression of *HvPap-10* in germinating barley seeds, alongside genes of other PLCP subgroups (*HvPap-4*, *HvPap-6*, and *HvPap-17*) [22]. Although the involvement of *PLCP* genes in barley physiological processes, such as leaf senescence, has been assessed using RT-qPCR [17], a comparative analysis of the protein levels of all active PLCP subfamilies in various barley organs during plant development has not been conducted. Moreover, gene expression profiles do not always correlate with proteomic changes and fail to reflect many functional aspects of the proteome [29].

The combination of activity-based protein profiling (ABPP) and mass spectrometric analysis is a powerful proteomic approach for studying protease function and may contribute to a comprehensive understanding of the role of various PLCP subfamilies in plant physiology [30]. Using DCG-04, a biotinylated derivative of E-64, an irreversibly pan-inhibitor of cysteine proteases, several PLCPs were detected in various plants [31,32,33]. The application of this probe enables the isolation (enrichment) of active PLCPs from complex proteomes, thereby increasing their concentration for subsequent detection via mass spectrometry (MS). Such enrichment reduces background noise generated by highly abundant proteins, ensuring more accurate quantification of low-abundance proteins that might otherwise remain undetected. Moreover, the ABPP method allows for the assessment of the functionally active population of enzymes, which may differ from their total abundance [34]. Since target proteins undergo enrichment, they generate a greater number of MS/MS spectra, which, in turn, enhances the reliability of quantitative analysis performed via spectral counting. Previously we conducted activity-based profiling of PLCPs during late-stage leaf senescence in barley [35]. In the present study we used this approach to evaluate the relative abundance of active PLCPs in various barley organs including roots, leaves, stems and seeds during different stages of plant development. The results obtained indicate that certain PLCPs are active only in specific barley organs or during specific stages of plant development.

## 2. Results and Discussion

### 2.1. Isolation and Identification of Active PLCPs

**Figure 1:**
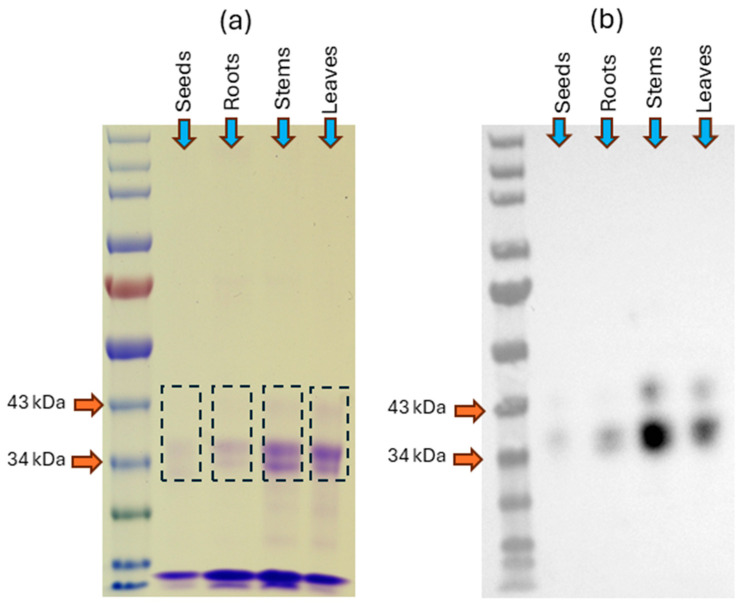
PLCPs were isolated from roots, leaves, and stems of 4-week-old barley plants, or from mature seeds, concentrated by ion-exchange chromatography, labeled with 10 μM DCG-04 for 3 h, and purified using streptavidin-agarose beads. Panel (**a**): PLCPs were separated by SDS-PAGE and visualized by Coomassie Blue staining. Dashed lines indicate gel slices excised for subsequent analysis by tandem MS. Panel (**b**): Proteins separated by SDS-PAGE and transferred to a nitrocellulose membrane were visualized using streptavidin-HRP and a chemiluminescent substrate.

Extracts from plant samples (shoots or roots of 4-day-old seedlings; roots, leaves, and stems of barley collected at different time points after sowing, and ungerminated seeds) were applied to a DEAE-Sepharose column and eluted using 1.5 M NaCl as elution buffer. Fractions containing active PLCPs (measured using the fluorogenic substrate Z-FR-AMC) were pooled and labeled with DCG-04, which binds to the active site of PLCPs [36]. It should be noted that the Z-FR-AMC cleavage activity of the pooled fractions was completely inhibited by 1 μM E-64, a specific cysteine protease inhibitor, confirming the presence of PLCPs [33]. The labeled proteins were captured on streptavidin-agarose beads. The column was washed with 1% SDS/1% NP-40 and bound proteins were eluted with Laemmli reducing sample buffer with excess biotin (25 mM) and heat [37]. Proteins were separated by SDS-PAGE and visualized by Coomassie Blue staining. Proteins were also transferred to nitrocellulose membrane and DCG-04-labeled proteases were detected using streptavidin-horseradish peroxidase (HRP) and a chemiluminescent substrate. A typical SDS-PAGE gel and a blot are shown in Figure 1. The two major bands (~43 and 38 kDa) were excised from the gel as a single gel slice and digested with trypsin, followed by peptide extraction and analysis by tandem MS.

Based on tandem MS analysis [38], twenty-three PLCPs belonging to nine known PLCP subfamilies were identified in protein extracts of barley samples collected at different developmental stages (Table 1). It should be noted that the THI1 subfamily, represented in barley by a single PLCP (UniProt ID: A0A8I6XRJ4) [35], was not detected in the analyzed samples.

The relative abundance of PLCPs was determined using the total number of spectral signals of their peptides obtained by data-dependent acquisition (DDA) as described previously in detail [35]. As an example, Supplementary Table S1 shows the values of “total spectrum count” and the fractions of PLCPs (in %) detected in mature (dry) seeds and samples from 7-week-old barley roots, leaves, and stems.

### 2.2. Relative Abundance of PLCP Classes in Barley Roots and Leaves/Shoots

**Figure 2:**
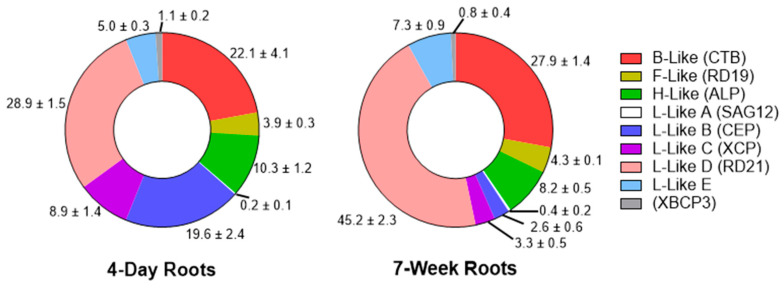
Relative abundance of PLCP subgroups in young barley roots (4th day of seed germination) and at the 7th week of plant development. Data are shown as fractions (percent of total “DCG-04 reactive” PLCPs). The data represent the means ± S.D. of three technical replicates.

In roots of 4-day-old seedlings, cathepsin B-like (CTB subfamily) and L-like D (RD21 subfamily) proteases accounted for the largest proportions (22.1% and 28.9%, respectively) of all PLCP classes, and their relative abundance increased during subsequent plant development (Figure 2). On the other hand, although the proportions of cathepsin L-like B (CEP subfamily) and L-like C (XCP subfamily) proteases were relatively high (19.6% and 8.9%, respectively) in roots of 4-day-old plants, the fraction of these proteases decreased in the roots of 7-week-old plants. The proportion of PLCPs from the other protease subfamilies in roots changed only slightly during barley development. It should be noted that the RD21A and CTB *Arabidopsis* orthologs were also among the major PLCPs identified in the apoplastic fluid of maize roots [33].

**Figure 3:**
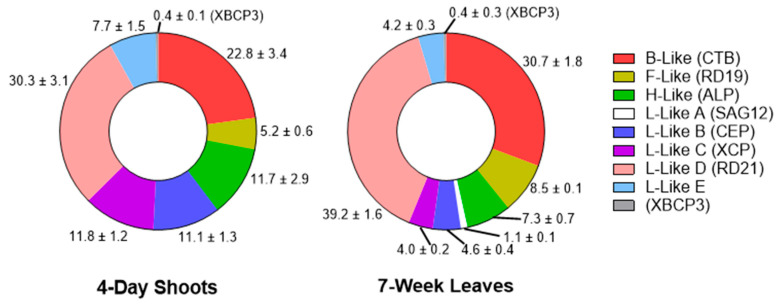
Relative abundance of PLCP subgroups in barley shoots on the 4th day of seed germination and in leaves on the 7th week of plant development. Data are shown as fractions (percent of total “DCG-04 reactive” PLCPs). The data represent the means ± S.D. of three technical replicates.

In shoots of 4-day-old seedlings, the proportions of B-like (CTB subfamily) (22.8%), L-like D (RD21 subfamily) (30.3%), L-like C (XCP subfamily) (11.8%), H-like (ALP subfamily) (11.7%), and L-like B (CEP subfamily) (11.1%) PLCPs were high (Figure 3). The other classes accounted for less than 10% each, including L-like E (7.7%), F-like (RD19 subfamily) (5.2%), and XBCP3 (0.4%). L-like A (SAG12 subfamily) proteases were not detected in shoots of 4-day-old seedlings. Similarly to roots, the proportions of B-like and L-like D proteases increased further in the leaves of developing plants, as compared to seedlings (Figure 3).

Below, the proportions of identified PLCPs from each of the nine subfamilies are examined in detail across barley organs (roots, leaves/shoots, stems, and seeds) and developmental stages.

#### 2.2.1. Cathepsin L-like D Proteases (RD21 Subfamily) in Roots and Leaves/Shoots

**Figure 4:**
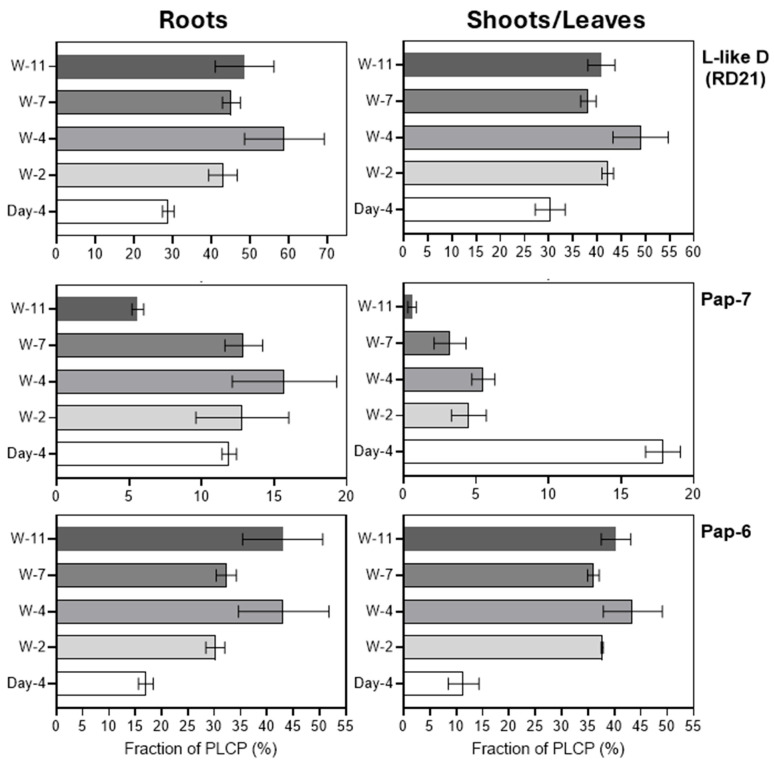
Relative abundance (in %) of the identified L-like D (RD21 subfamily) proteases in roots (left panels) and leaves/shoots (right panels) at different stages of barley development. Separate panels show the proportion of HvPap-6, HvPap-7, and total L-like D-proteases. The total content of L-like D-proteases was calculated as the sum of HvPap-6 and HvPap-7 proportions. The data represent the means ± S.D. of three technical replicates.

Two of three previously described barley L-like D proteases, HvPap-6 and HvPap-7, were detected during root and leaf development. Based on total spectral counts, HvPap-6 accounted for 17.0–43.0% of all PLCPs in roots, and 11.4–43.5% in leaves/shoots (Figure 4). The proportion of HvPap-6 increased in roots and leaves of 2-week-old plants, remaining at approximately the same level throughout subsequent plant development (2–11 weeks). In contrast, the proportion of HvPap-7 decreased in leaves as early as the 2nd week and reached a minimum at the terminal stage (11 weeks), whereas in roots, a decrease in the proportion of this enzyme was observed only at the terminal stage (Figure 4). Thus, the relative increase in L-like D-proteases during root and leaf development was due to the increase in the proportion of HvPap-6.

#### 2.2.2. Cathepsin B-like Proteases (CTB Subfamily) in Roots and Leaves/Shoots

**Figure 5:**
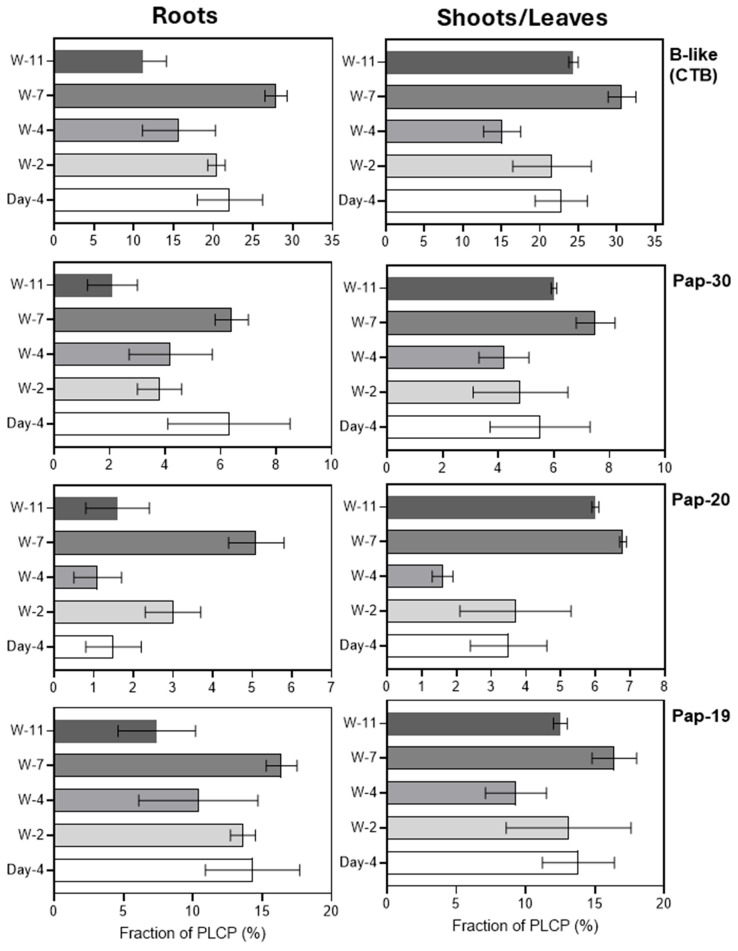
Relative abundance (in %) of the identified B-like (CTB subfamily) PLCPs in roots (left panels) and leaves/shoots (right panels) at different stages of barley development. Separate panels show the proportions of HvPap-19, HvPap-20, HvPap-30, and the total content of B-like proteases calculated as the sum of the proportions of HvPap-19, HvPap-20, and HvPap-30. The data represent the means ± S.D. of three technical replicates.

All three previously described barley B-like proteases, including HvPap-19, HvPap-20, and HvPap-30, were detected during root and leaf development, with HvPap-19 accounting for the largest fraction (7.4–16.4% in roots and 9.3–16.4% in leaves/shoots, respectively). The total fraction, calculated as the sum of all three of these proteases, increased slightly in roots and leaves from weeks 2 to 7, and then decreased in roots at week 11. At week 11, a decrease in the content of the HvPap-19 and HvPap-30 fractions was observed in the roots, whereas at week 4, a decrease in the HvPap-20 fraction was noted in the leaves (Figure 5).

#### 2.2.3. Cathepsin H-like Proteases (ALP Subfamily) in Roots and Leaves/Shoots

**Figure 6:**
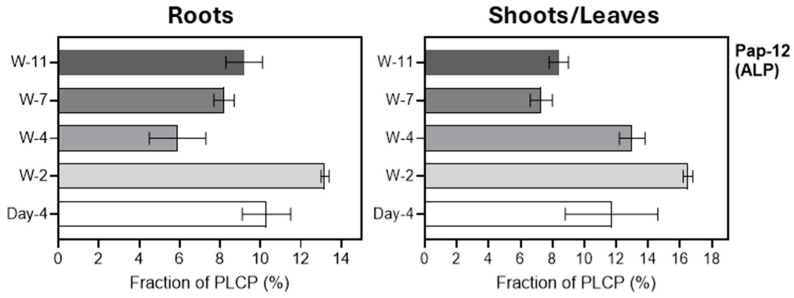
Relative abundance (in %) of HvPap-12 in roots (left panel) and leaves/shoots (right panel) at different stages of barley development. The data represent the means ± S.D. of three technical replicates.

Barley H-like proteases are represented by a single protease, HvPap-12 (aleurain). The overall pattern of changes in this protease was similar in roots and leaves, reaching a maximum in two-week-old samples (Figure 6). Although aleurain was historically first discovered in the aleurone layer of barley grain [39], its role in the development of barley roots and leaves requires further evaluation.

#### 2.2.4. Cathepsin F-like Proteases (RD19 Subfamily) in Roots and Leaves/Shoots

HvPap-1 protease, a member of the F-like subfamily, was detected during root and leaf development, although two other previously described proteases from this family, HvPap-2 and HvPap-3, were not found.

**Figure 7:**
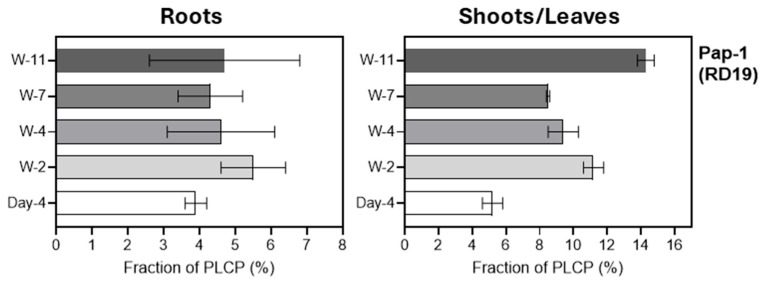
The relative abundance (in %) of HvPap-1, belonging to the F-like (RD19 subfamily) proteases in roots (left panel) and leaves/shoots (right panel) at different stages of barley development. The data represent the means ± S.D. of three technical replicates.

The overall pattern of changes in HvPap-1 was similar to HvPap-12, increasing from 4-day-old to 2-week-old plants. The highest Pap-1 proportion was found in leaves of 11-week-old barley plants (Figure 7). Previously HvPap-1 was reported to play an essential role in protein mobilization during seed germination [40]. The overexpression of HvPap-1 increased germination rate, while silencing this gene resulted in a phenotype with increased starch amounts in seeds [41]. RD19 protease, the *Arabidopsis* ortholog of HvPap-1, is associated with pathogen defense, whereas its other orthologs act as modulators in plant signaling pathways [42].

#### 2.2.5. Cathepsin L-like C Proteases (XCP Subfamily) in Roots and Leaves/Shoots

**Figure 8:**
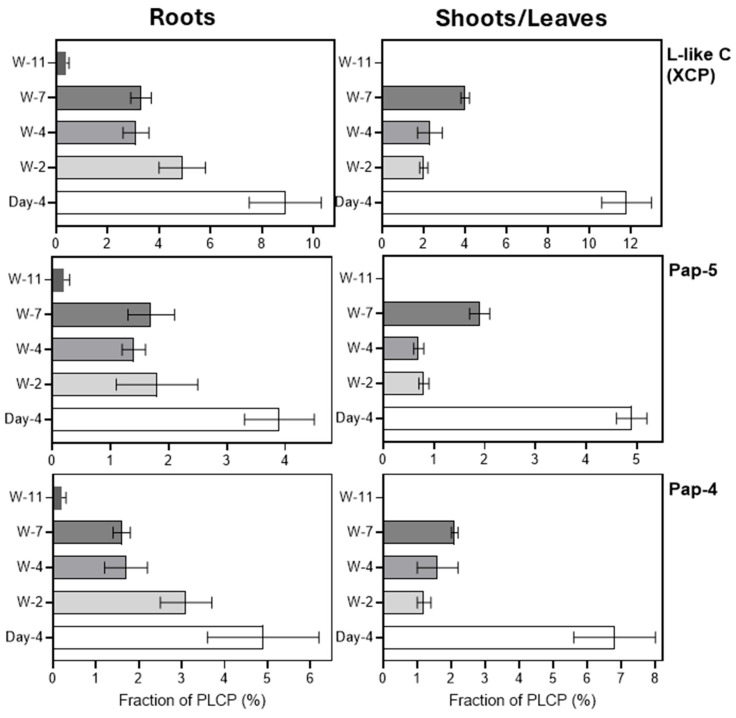
Relative abundance (in %) of the identified L-like C (XCP subfamily) PLCPs in roots (**left panels**) and leaves/shoots (**right panels**) at different stages of barley development. Separate panels show the proportions of HvPap-4, HvPap-5, and the total content of L-like C proteases calculated as the sum of the proportions of HvPap-4, HvPap-5, and HvPap-26. The data represent the means ± S.D. of three technical replicates.

All three previously described L-like C proteases, including HvPap-4, HvPap-5, and HvPap-26, were detected during root development, although HvPap-26 was only present at a low proportion (~0.1%) in shoots and roots of 4-day-old seedlings. The proportions of HvPap-4 and HvPap-5 and their sum decreased between 4-day-old seedlings and older plants, both in roots and leaves/shoots (Figure 8). These data are consistent with the high level of *HvPap-4* gene expression in germinating seeds and 7-day-old seedlings, identified in the study by Martinez et al. [22], and indicate an important role for proteases of this subfamily during the seedling stage of barley development (see also Section 2.3).

#### 2.2.6. HvPap-8 Protease (XBCP3 Subfamily) in Roots and Leaves/Shoots

The XBCP3 subfamily is represented by HvPap-8 in barley. The proportion of this protease in all analyzed samples, including samples of roots, leaves, and shoots, was relatively low (0.4–1.3%) (Table 2).

#### 2.2.7. Cathepsin L-like B Proteases (CEP Subfamily) in Roots and Leaves/Shoots

Four (HvPap-9, HvPap-10, HvPap-14, and HvPap-42) of the seven known members of the cathepsin L-like B protease subfamily were detected in the shoots and roots of 4-day-old seedlings (19.6% and 11.1%, respectively), with HvPap-10 being the most abundant. Notably, only HvPap-14 was detected in all root and leaf samples, including those obtained from mature plants, whereas HvPap-9, HvPap-10, and HvPap-42 proteases were identified exclusively in seedlings (Table 3). These results are consistent with the embryo-specific expression pattern of HvPap-10 established in the study by Martinez et al. [22].

HvPap-14 protease has previously been reported to be involved in the degradation of the large subunit of ribulose-1,5-bisphosphate carboxylase/oxygenase (Rubisco) in leaf chloroplasts [9], but the role of this protease in barley roots remains unknown. In *Arabidopsis*, three orthologs (AtCEP1, AtCEP2, and AtCEP3) of L-like B proteases (CEP subfamily) have been identified and shown to act in a tissue- and organ-specific fashion during seedling and root development [43,44]. Among them, AtCEP2 is involved in the elongation of the primary root in *Arabidopsis* [45]. Moreover, the first barley cysteine protease described as participating in the proteolytic degradation of grain storage proteins was the cathepsin L-like B protease named EP-B [28]. According to the existing classification, barley proteases EP-B1 and EP-B2 (UniProt ID: P25249 and P25250) correspond to HvPap-10, and barley EP-A is HvPap-9 (UniProt ID: O04677). These proteases are orthologues of gliadain, a gibberellin-induced cysteine protease found in germinating wheat (*Triticum aestivum* L.) seeds [46]. Thus, our findings support an important role for L-like B proteases in early plant development (see also Section 2.4, regarding the distribution of PLCPs in barley seeds).

#### 2.2.8. Cathepsin L-like E Proteases in Roots and Leaves/Shoots

Based on genetic analysis, sixteen L-like E proteases have been described in barley [17]. However, in root and shoot/leaf samples, only seven members of this subfamily were detected, including HvPap-13, HvPap-16, HvPap-22, HvPap-27, HvPap-28, HvPap-29, and HvPap-35. Interestingly, HvPap-27 (0.4–1.2%), HvPap-28 (1.5%), HvPap-29 (1.4–4.1%), and HvPap-35 (0.4%) were detected only in roots, whereas HvPap-16 (0.2–1.1%) and HvPap-22 (0.4%) were found exclusively in leaves. It should be noted that the *HvPap-16* gene was also expressed exclusively in leaves [22]. HvPap-13 was detected in all root and leaf/shoot samples, whereas HvPap-29 was detected only in all root samples (Table 4). Since orthologs of the L-like E subfamily may only be present in grasses (Poaceae) [35], their analysis in the context of plant development and evolution is of particular interest.

#### 2.2.9. Cathepsin L-like A Proteases (SAG12 Subfamily) in Roots and Leaves/Shoots

Barley cathepsin L-like A proteases are represented by a single protease, HvPap-17. The proportion of this protease reached its maximum level in the fourth week of root and leaf development (2.0 and 3.3%, respectively) and then declined (Table 5). Otegui et al. reported that a homozygous *SAG12 Arabidopsis* mutant does not show any visually detectable phenotypical alteration during senescence [47], suggesting that SAG12 is functionally redundant with other proteases. The role of HvPap-17 (an ortholog of SAG12) in barley senescence and development remains to be determined.

### 2.3. PLCP Expression in Stems Compared to Leaves

**Figure 9:**
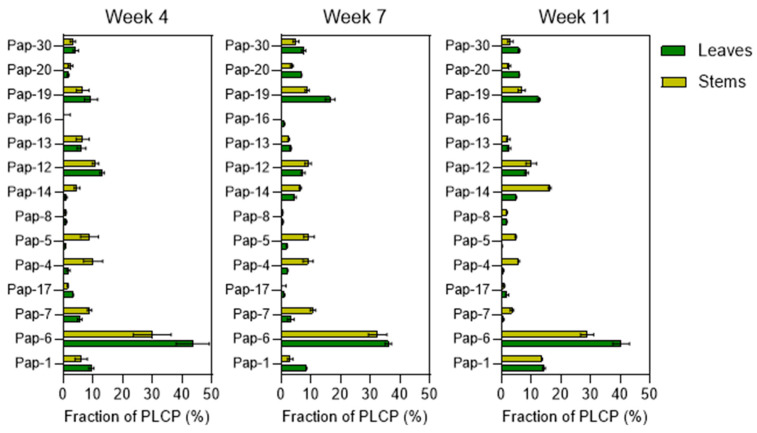
The relative abundance (in %) of identified PLCPs in leaves and stems at different stages of barley development. The data represent the means ± S.D. of three technical replicates.

The distribution (in %) of PLCPs in stems compared to leaves for each time point is shown in Figure 9. The largest difference was found for the cathepsin L-like C (XCP subfamily) proteases HvPap-4 and HvPap-5. Their proportion was higher in stems than in leaves of 4-week, 7-week, and 11-week-old plants (6–12, 4–5, and 10–20 times higher, respectively, compared to leaf samples harvested at the same time points). This is consistent with the important role of orthologs of these proteases in cellular autolysis of tracheal elements in *Arabidopsis* [48,49] and *Fabaceae* [50]. In all stem samples of barley, a higher proportion of HvPap-14 and HvPap-7, belonging to the cathepsin L-like B (CEP subfamily) and L-like D (RD21 subfamily) subfamilies, respectively, was also observed. Conversely, a lower proportion of HvPap-19, HvPap-20, and HvPap-30, belonging to cathepsin B-like proteases (CTB subfamily), was observed in stems compared to leaves in 7-week and 11-week samples.

### 2.4. PLCPs in Seeds

**Figure 10:**
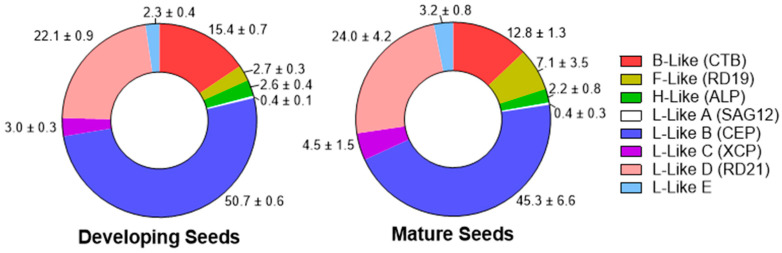
The relative abundance of PLCPs (in %) in developing and mature (dry) barley seeds. The data represent the means ± S.D. of three technical replicates.

Cathepsin L-like D (RD21 subfamily) (22.1–24.0%), L-like B (CEP subfamily) (45.3–46.0%), and B-like (CTB subfamily) (12.8–15.4%) proteases are the principal detected PLCP subgroups in seeds (Figure 10). Although the total proportion of L-like B proteases in developing (immature) and mature (dry) seeds was approximately the same, the relative abundance of HvPap-10 increased, while HvPap-14 decreased, during seed maturation (Supplementary Table S2). It should be noted that AtCEP1, an ortholog of these proteases, has previously been reported to be involved in pollen and seed development [44,51].

Our results show that cathepsin L-like D-proteases represent a large fraction of all active PLCPs in all barley organs throughout plant development, including roots, leaves, stems, and seeds. Previously, *triticain α* (a wheat ortholog of *HvPap-6*) expression was detected during seed imbibition and early germination [52]. Our findings are also consistent with recent results by Havé et al. [32] showing that triticain α is the major PLCP in naturally senescent wheat leaves. The highest number of spectral counts in maize apoplastic fluid was recorded for CP1A and CP1B, orthologs of *Arabidopsis* RD21A [53]. Furthermore, Sekhon et al. [54] demonstrated the functional importance of Mir3 (a HvPap-6 ortholog) for leaf senescence. Those authors also showed that knockout of *Arabidopsis* RD21A, a HvPap-6 ortholog, leads to a delayed-senescence phenotype. Protease V7CU09 (RD21A ortholog) was also the major PLCP in leaves of *Phaseolus vulgaris* (French bean) [55].

In the present study, 23 cysteine proteases, out of 42 previously described barley PLCPs [17], were identified as active in protein extracts from barley samples collected at various stages of development. Thus, we were able to identify 12 additional barley PLCPs compared to the data obtained for barley leaves at a late stage of senescence (~14 weeks of age) [35]. Most additional proteases were active in roots or 4-day-old seedlings, including HvPap-9, -10, -22, -26, -27, -28, -29, -35, and -42 (Table 1). While newly identified HvPap-4, -5, and -30 were active in leaves of developing plants up to week 7 (HvPap-4 and -5) or week 11 (HvPap-30), they were no longer detected in late-senescing leaves [35]. PLCPs of the cathepsin B-like subfamily, such as HvPap-30, are characterized by a high number of disulfide bridges compared to other PLCPs [3,56], which may make them more sensitive to inactivation at late senescence stages.

A limitation of this study is that only a single biological replicate was analyzed for each sample type, although each sample comprised a substantial amount of plant material (~70 g; Section 3.3). By contrast, three technical replicates were analyzed per sample, as indicated in the figure legends. Furthermore, all plants were grown under controlled conditions (Section 3.2). Consequently, the data do not capture biological variation in the relative abundances of the identified proteases, nor do they provide insight into the relative importance of these proteases under field conditions shaped by biotic and abiotic stresses.

A previously conducted analysis of the barley genome enabled the identification of up to 41 members of the PLCP family (HvPap-1 to HvPap-42; notably, HvPap-10 and HvPap-11 represent distinct allelic variants of the same gene) [17]. There are several possible reasons why some PLCPs were not detected in the present study. This may be attributed to low activity which, in turn, may be due to transcriptional or post-transcriptional regulation including protein stability and activation status.

To test the hypothesis that some PLCPs were not detected due to low or absent transcription of their genes, we extracted expression data for each *PLCP* gene from the BarleyExpDB database [57]. The expression of each *PLCP* gene in leaves, roots, and germinated seeds was compared with data on the relative abundance of active PLCPs obtained from corresponding barley samples within the framework of the present study. *HvPap-23*, *-31*, *-32*, and *-39* were not included in the analysis, as their gene identifiers were not found in the latest variety ‘Morex’ genome assemblies (versions V2 or V3). It should be noted that these four PLCPs were also not detected via tandem MS in any of the samples analyzed in this study.

**Figure 11:**
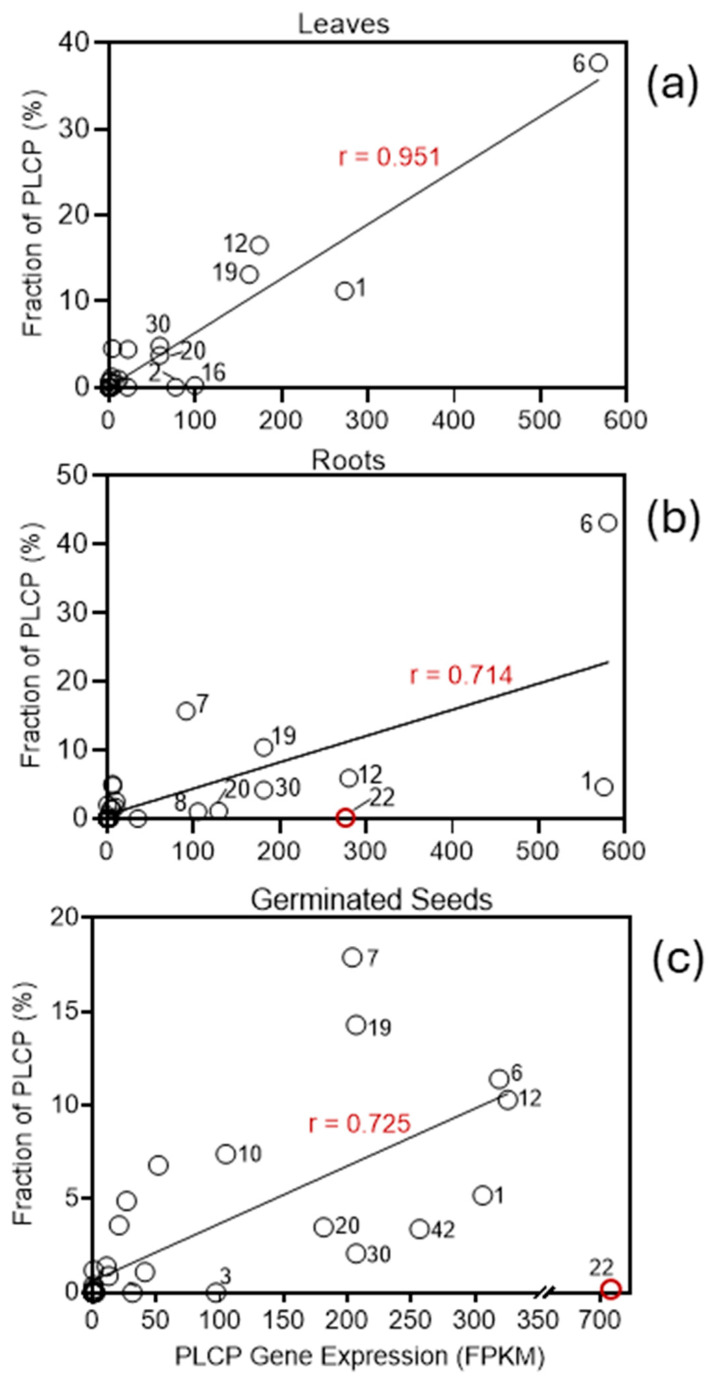
Correlations between the relative abundances of active PLCPs (%) and *PLCP* gene expression in barley leaves (**Panel a**), roots (**Panel b**), and germinated seeds (**Panel c**) based on ABPP (this study) and RNA-sequencing data, extracted from the BarleyExpDB database. The expression of each *PLCP* gene is expressed as FPKM (fragments per kilobase of transcript per million mapped reads) values. FPKM normalizes raw counts for both sequencing depth and gene length, making it appropriate for within-sample comparisons [59]. Each number (N) on the panels denotes a PLCP name in the format HvPap-N; the names correspond only to proteases with a relatively high level of gene or protein expression. HvPap-22, marked with a red circle, was excluded from the regression calculation. For the “Leaves” category, protein expression levels (expressed as a percentage of the total number of “DCG-04-reactive” PLCPs) from 2-week-old samples were used; for the “Roots” category, protein expression levels corresponded to samples from 4-week-old plants; and for the “Germinated Seeds” category, values were taken as the arithmetic mean of the values for the roots and shoots of 4-day-old seedlings.

The results of this analysis indicate that the relative abundance of active PLCPs correlates well with the expression of *PLCP* genes in barley leaves (r = 0.904), although Pearson linear correlation coefficients (calculated excluding one data point, HvPap-22) for roots and germinating seeds were lower (r = 0.714 and 0.725, respectively; Figure 11). Based on a fixed FPKM threshold of 1 [58], expression of nineteen *PLCP* genes (*HvPap-3, -9*, *-10*, *-15*, *-24–29*, *-33–38*, and *-40–42*) was not detected in barley leaves (Panel a), eighteen *PLCP* genes (*HvPap-3*, *-9*, *-10*, *-15*, *-16*, *-21*, *-24–26*, *-33–38*, and *-40–42*) in barley roots (Panel b), and twelve *PLCP* genes (*HvPap-16*, *-21*, *-24*, *-25*, *-28*, *-33–36*, *-40*, and *41*) in germinated barley seeds (Panel c). These HvPaps are localized at the origin of the graphs, reflecting the absence of both detectable gene expression and active PLCPs by ABPP (Figure 11). Active HvPap-22 was detected in the shoots of 4-day-old seedlings at very low levels and was absent from the roots of mature plants, despite the relatively high level of expression of the corresponding gene in the germinated seeds and roots. The reason for the absence of active HvPap-22 in the roots requires further investigation.

Since six PLCPs of the cathepsin L-like E subfamily (HvPap-16, -21, -25, -33, -34, and -36) and three PLCPs of the cathepsin L-like B subfamily (HvPap-24, -40, and -41) were not detected at either the gene transcription or protein/activity level in any of the analyzed samples, the corresponding genes may be inactive. In addition, expression of some PLCPs may be limited to specialized tissues or organs, such as pollen or other reproductive structures that were not analyzed here. Some genes annotated as PLCPs may not encode functional proteases capable of interacting with the utilized activity probe. In plants, gene duplication is a relatively common phenomenon; often, only one of the copies retains its original function, while the other either loses function or acquires new functions distinct from the original ones [60,61]. Finally, some previously annotated protease genes may be present in other barley varieties but may be absent or nonfunctional in the variety ‘Gemcraft’ used here.

## 3. Materials and Methods

### 3.1. Materials and Reagents

E-64 (trans-epoxysuccinyl-L-leucylamido-(4-guanidino)butane), Z-Phe-Arg-7-amido-4-methylcoumarin (Z-FR-AMC), DEAE-Sepharose CL-6B, and dithiothreitol (DTT) were from Millipore-Sigma (St. Louis, MO, USA). The color-coded prestained protein marker was from Cell Signaling Technologies (Danvers, MA, USA). Dimethyl sulfoxide (DMSO) was from Acros Organics (Fair Lawn, NJ, USA). DCG-04, a biotinylated E-64 derivative [36], was obtained from Psyclo Peptide Inc. (Shanghai, China). High-capacity streptavidin agarose resin was from Thermo Fisher Scientific (Rockford, IL, USA).

### 3.2. Plant Material

Barley plants (*Hordeum vulgare* L. var. ‘GemCraft’) [62] were grown as described previously [63]. Briefly, barley plants (three to five plants per 4 L pot) were grown in potting soil in a climate-controlled growth room set at 22 °C and a humidity of ~ 60%. The growth room lights were Philips Greenpower toplighting 200–400 v LED rails providing between 300 and 500 µmol m^− 2^ s^− 1^ photosynthetically active radiation at the level of growing plants, with 16 h/8 h day-night cycles maintained. Plants were fertilized once per week until flowering with Peter’s Professional General-Purpose fertilizer (250 mL per pot; 4 g/L; Scotts-Sierra Horticultural Products Company, Marysville, OH, USA).

The five to seven uppermost leaf blades were sampled at 4, 7, and 11 weeks after sowing. Additionally, at week 2, the entire shoot system was harvested (serving as the leaf sample for that week). Root systems were sampled 2, 4, 7, and 11 weeks after sowing (on the same days as the leaves), after which they were cleaned of soil using distilled water. Stems were sampled from plants at 4, 7, and 11 weeks of age. Stem sampling began at week 4, as at week 2, the plant was in the tillering phase of development [64]. Seeds were germinated between moistened sheets of Whatman filter paper in the dark at room temperature. On the fourth day, the shoots and roots of the germinated seeds were harvested. Developing seeds were collected from 11-week-old plants (4 weeks after the onset of flowering); mature/dry seeds were collected at the late maturation stage (from plants aged ~15 weeks) [65] and subsequently stored for up to one year at room temperature. All plant samples were first frozen in liquid nitrogen, then powdered and stored at −80 °C until protein extraction.

### 3.3. Ion-Exchange and Affinity Purification of PLCPs

Due to the labor-intensive nature of downstream analyses, experiments were performed using single, large-scale extracts. The use of substantial amounts of starting material ensured that each sample was representative of the respective organ and developmental stage. Each sample, except for 4-day-old seedlings and 2-week-old plants, was obtained from 5–6 plants; samples of two-week-old plants were obtained from ~18–20 plants. The required amount of material (~70 g) from 4-day-old seedlings was obtained from a large number of seedlings. Thus, soluble proteins were extracted by homogenizing ~70 g of biological material in 350 mL of ice-cold 50 mM Tris-HCl buffer (pH 7.4). Homogenates were filtered through one layer of Miracloth (Calbiochem, San Diego, CA, USA), centrifuged at 20,000× *g* for 20 min at 4 °C, and the supernatants were then filtered through a 0.45 μm filter (Avantor, Radnor, PA, USA). Proteins were separated using anion-exchange chromatography on a DEAE-Sepharose CL-6B column (volume 10 mL) equilibrated with 50 mM Tris-HCl buffer (pH 7.4). The column was washed with equilibration buffer, and bound material was eluted with 1.5 M NaCl. Collected fractions (1 mL each) were kept in an ice bath and analyzed for peptidase activity with a fluorogenic substrate, Z-FR-AMC (Section 3.4). Pooled activity was completely inhibited by E-64 (100 nM final concentration), a specific inhibitor of cysteine proteases. Fractions with active cysteine proteases were pooled. To reduce the NaCl concentration and exchange the buffer to 0.1 M Na-citrate (pH 5.5), three sequential filtration steps using 10-kDa ultrafiltration 15-mL units (Merck Millipore Ltd., Carrigtwohill, County Cork, Ireland) were utilized, and the samples were then incubated with gentle shaking with 10 µM DCG-04 in 0.1 M Na-citrate buffer (pH 5.5) in the presence of 2 mM DTT for 3 h at 37 °C. No remaining PLCP activity was found after three hours, as confirmed using the Z-FR-AMC fluorogenic substrate [63]. After the incubation, unbound DCG-04 was removed, and the buffer was exchanged to 50 mM Tris-HCl buffer (pH 7.4) by three sequential filtration steps using 10-kDa ultrafiltration 15-mL units.

Affinity enrichment of DCG-04-reactive PLCPs was performed using streptavidin-agarose. Each sample was eluted through 120-μL bed volume of streptavidin-agarose at room temperature. To reduce nonspecific binding, the column was washed with 1% SDS and 1% NP-40 in 50 mM Tris-HCl buffer (pH 7.4). After washing, bound polypeptides were eluted by adding 100 µL of Laemmli reducing sample buffer with excess biotin (25 mM) and boiling for 7 min [37].

DCG-04-reactive proteins were then separated on three distinct lanes of ready-made ExpressPlusTM 4–12% acrylamide gels (GenScript Inc., Piscataway, NJ, USA) in Tris-MOPS SDS-PAGE Running Buffer (GenScript), producing three technical replicates of each sample for following analyses. The gels were fixed for 45 min, stained with Coomassie Brilliant Blue R-250 overnight, and destained with 50% methanol, 10% acetic acid, and 40% water. Visible bands from the Coomassie-stained gels were excised (Figure 1) and shipped to the IDeA National Resource for Quantitative Proteomics at the University of Arkansas (Little Rock, AR, USA).

### 3.4. Enzymatic Assays and Protein Determination

The fluorogenic substrate Z-FR-AMC was used to measure PLCP activity. The emitted fluorescence was detected with a SpectraMax M2 microplate reader (Molecular Devices, San Jose, CA, USA) with λ_ex_ = 360 nm and λ_em_ = 460 nm. Before use, the substrates were dissolved in DMSO at 10 mM and stored at −20 °C. The measurements were made in 96-well black microplates (PerkinElmer Inc, Waltham, MA, USA) at room temperature, and each well contained a 100 μL final volume of 0.1 M Na-citrate buffer (pH 5.5) with DTT (2 mM), an aliquot of extract or chromatographic fraction, and fluorogenic substrate (25 μM). The reaction was initiated by the addition of the substrate. The final concentration of DMSO in microplate wells was 1% in all assays. The assays were conducted at room temperature, and relative fluorescence readings were recorded over a period of 10 min.

### 3.5. Gel-Based Tandem MS Analysis

SDS-PAGE gel bands were subjected to in-gel trypsin digestion [66]. Gel segments were destained in 50% methanol with 50 mM ammonium bicarbonate, followed by reduction in 10 mM Tris[2-carboxyethyl]phosphine (Pierce, Rockford, IL, USA) and alkylation in 50 mM iodoacetamide (Sigma-Aldrich, St. Louis, MO, USA). Gel slices were then dehydrated in acetonitrile (Fisher, Rockford, IL, USA), followed by addition of 100 ng porcine sequencing-grade modified trypsin (Promega, Madison, WI, USA) in 50 mM ammonium bicarbonate (Sigma-Aldrich) and incubation at 37 °C for 12–16 h. Peptide products were then acidified in 0.1% formic acid (Pierce).

Tryptic peptides were separated by reverse phase XSelect CSH C18 2.5 µm resin (Waters, Milford, MA, USA) on an in-line 150 × 0.075 mm column using a nanoAcquity UPLC system (Waters). Peptides were eluted using a 60 min gradient from a 98:2 to 65:35 buffer A:B ratio (buffer A = 0.1% formic acid, 0.5% acetonitrile; buffer B = 0.1% formic acid, 99.9% acetonitrile). Eluted peptides were ionized by electrospray (2.4 kV) followed by tandem MS analysis using higher-energy collisional dissociation (HCD) on an Orbitrap Fusion Tribrid mass spectrometer (Thermo Fisher Scientific) in top-speed data-dependent mode. MS data were acquired using the Fourier transform mass spectrometry (FTMS) analyzer in profile mode at a resolution of 240,000 over a range of 375 to 1500 *m*/*z*. Following HCD activation, tandem MS data were acquired using the ion trap analyzer in centroid mode and normal mass range with precursor mass-dependent normalized collision energy between 28.0 and 31.0.

### 3.6. Database Searching and Criteria for Protein Identification

Peptide search results were analyzed with Scaffold DDA version 6.7.4 (Proteome Software Inc., Portland, OR, USA). Peptide and protein thresholding, and protein grouping were performed by Scaffold DDA version 6.8.0. Peptide and protein results were loaded from Mascot version 2.6.2 (Matrix Science, London, UK). Mascot was set up to search the 2026_01 UniProt *Hordeum_vulgare* database, assuming the digestion enzyme trypsin. Peptide identifications were subsequently thresholded to achieve a peptide FDR (a false discovery rate) better than 1.0% based on q-values computed from the Mascot scores. Proteins that contained similar peptides and could not be differentiated based on MS/MS analysis were grouped to satisfy the principles of parsimony. Protein groups with a minimum of 2 identified peptides were thresholded to achieve a protein FDR better than 1.0% based on q-values. Normalization was applied to TIC (a total ion chromatogram) and exclusive TIC. Protein probabilities were assigned by the Protein Prophet algorithm [67].

## 4. Conclusions

In shoots and roots of 4-day-old seedlings, B-like and L-like D proteases accounted for the largest proportions among all active PLCP classes, and their relative abundance increased further during subsequent plant development in roots and leaves, respectively. On the other hand, although the proportions of L-like B and L-like C proteases were relatively high in shoots and roots of seedlings, the fraction of these proteases decreased in the roots and leaves of developing plants, because HvPap-9, HvPap-10, and HvPap-42 were only detected in seedlings, while HvPap-14 was also detected in roots and leaves during subsequent developmental stages. These data suggest that L-like B and L-like C proteases play important roles in seedling development. Moreover, our data are consistent with known information regarding the role of EP-A and EP-B, representatives of the L-like B subfamily, in the proteolysis of storage proteins [4,27,68]. Organ-specific active PLCPs were also found: HvPap-26, belonging to the L-like C proteases, was present in the shoots and roots of seedlings albeit at low proportions; PLCPs of the L-like E subfamily, including HvPap-27, HvPap-28, and HvPap-29, were detected only in roots, whereas HvPap-16 was found exclusively in leaves. Thus, the results obtained indicate that specific PLCPs are active only in certain barley organs and allow for a quantitative assessment of how their relative abundance in these organs changes during plant development.

## Figures and Tables

**Table 1:** PLCPs detected by tandem MS in different barley samples.

Subfamily	PLCP Name	UniProt ID	Plant Organ
F-like (RD19)	HvPap-1	F2DDC9	Roots, leaves, stems, seeds
L-like D (RD21)	HvPap-6	A0A8I6WYU4	Roots, leaves, stems, seeds
	HvPap-7	F2E6V2	Roots, leaves, stems, seeds
L-like A (SAG12)	HvPap-17	A0A8I6Y6A5	Roots, leaves, stems, seeds
L-Like C (XCP)	HvPap-4	B4ESE6	Roots, leaves, stems, seeds
	HvPap-5	B4ESE7	Roots, leaves, stems, seeds
	HvPap-26	A0A287UKW8	Shoots and roots of seedlings
(XBCP3)	HvPap-8	B4ESF0	Roots, leaves, stems, seeds
L-like B (CEP)	HvPap-9	A0A8I6XX70	Shoots and roots of seedlings
	HvPap-10	A0A8I6XRW9	Shoots and roots of seedlings
	HvPap-42	A0A8I7BAT3	Shoots and roots of seedlings
	HvPap-14	B4ESF2	Roots, leaves, stems, seeds
H-like (ALP)	HvPap-12	A0A8I6Y1I6	Roots, leaves, stems, seeds
L-like E	HvPap-13	A0A8I6XJP4	Roots, leaves, stems, seeds
	HvPap-16	A0A8I6YT54	Shoots of seedlings, leaves, stems, seeds
	HvPap-22	A0A8I6WPU1	Shoots of seedlings
	HvPap-27	A0A8I6WQD4	Roots of seedlings
	HvPap-28	A0A8I6XSH6	Roots of 2-week plants
	HvPap-29	A0A8I7B9F2	Roots
	HvPap-35	A0A8I6WXF8	Roots of 2-week plants
B-like (CTB)	HvPap-19	A0A8I7BBL8	Roots, leaves, stems, seeds
	HvPap-20	A0A8I7B6S7	Roots, leaves, stems, seeds
	HvPap-30	A0A8I6X9J3	Roots, leaves, stems, seeds

**Table 2:** The relative abundance (in %) of HvPap-8 in roots and leaves/shoots at different stages of barley development.

Organ	4-Day Seedlings	Developing Plant			
		Week 2	Week 4	Week 7	Week 11
Roots	1.1 ± 0.2	1.3 ± 0.04	1.0 ± 0.5	0.8 ± 0.4	0.4 ± 0.1
Shoots or Leaves	0.4 ± 0.1	0.4 ± 0.1	0.9 ± 0.3	0.4 ± 0.3	1.8 ± 0.1

The data represent the means ± S.D. of three technical replicates.

**Table 3:** The relative abundance (in %) of the identified L-like B proteases (CEP subfamily) in roots and leaves/shoots at different stages of barley development.

PLCP Name	Organ	4-Day Seedlings	Developing Plant			
			Week 2	Week 4	Week 7	Week 11
HvPap-9	Roots	0.5 ± 0.3	N.D.	N.D.	N.D.	N.D.
HvPap-10		14.5 ± 2.3	N.D.	N.D.	N.D.	N.D.
HvPap-42		3.4 ± 0.5	N.D.	N.D.	N.D.	N.D.
HvPap-14		1.2 ± 0.2	1.5 ± 0.1	1.5 ± 0.8	2.6 ± 0.6	7.5 ± 0.4
Total L-like B		19.6 ± 2.4	1.5 ± 0.1	1.5 ± 0.8	2.6 ± 0.6	7.5 ± 0.4
HvPap-9	Shoots or Leaves	0.9 ± 0.3	N.D.	N.D.	N.D.	N.D.
HvPap-10		7.4 ± 1.2	N.D.	N.D.	N.D.	N.D.
HvPap-42		1.8 ± 0.2	N.D.	N.D.	N.D.	N.D.
HvPap-14		1.0 ± 0.3	0.6 ± 0.2	0.9 ± 0.4	4.6 ± 0.4	5.0 ± 0.1
Total L-like B		11.1 ± 1.3	0.6 ± 0.2	0.9 ± 0.4	4.6 ± 0.4	5.0 ± 0.1

N.D., signal not detected. The data represent the means ± S.D. of three technical replicates.

**Table 4:** The relative abundance (in %) of the identified cathepsin L-like E proteases in roots and leaves/shoots at different stages of barley development.

PLCP Name	Organ	4-Day Seedlings	Developing Plant			
			Week 2	Week 4	Week 7	Week 11
HvPap-13	Roots	3.2 ± 0.1	2.8 ± 0.6	4.8 ± 1.3	1.6 ± 0.6	3.6 ± 0.1
HvPap-16		N.D.	N.D.	N.D.	N.D.	N.D.
HvPap-22		N.D.	N.D.	N.D.	N.D.	N.D.
HvPap-27		0.4 ± 0.1	1.2 ± 0.3	N.D.	1.2 ± 0.4	1.0 ± 0.2
HvPap-28		N.D.	1.5 ± 0.1	N.D.	N.D.	8.5 ± 1.9
HvPap-29		1.4 ± 0.3	4.1 ± 0.3	2.5 ± 1.1	4.5 ± 0.5	5.0 ± 0.7
HvPap-35		N.D.	0.4 ± 0.2	N.D.	N.D.	N.D.
Total L-like E		5.0 ± 0.3	10.0 ± 0.8	7.3 ± 1.7	7.3 ± 0.9	18.1 ± 2.0
HvPap-13	Shoots or Leaves	7.1 ± 1.5	4.4 ± 0.8	6.1 ± 1.5	3.1 ± 0.3	2.5 ± 0.6
HvPap-16		0.2 ± 0.05	0.2 ± 0.1	N.D.	1.1 ± 0.1	N.D.
HvPap-22		0.4 ± 0.1	N.D.	N.D.	N.D.	N.D.
HvPap-27		N.D.	N.D.	N.D.	N.D.	N.D.
HvPap-28		N.D.	N.D.	N.D.	N.D.	N.D.
HvPap-29		N.D.	N.D.	N.D.	N.D.	N.D.
HvPap-35		N.D.	N.D.	N.D.	N.D.	N.D.
Total L-like E		7.7 ± 1.5	4.6 ± 0.8	6.1 ± 1.5	4.2 ± 0.3	2.5 ± 0.6

N.D., signal not detected. The data represent the means ± S.D. of three technical replicates.

**Table 5:** The relative abundance (in %) of the identified HvPap-17 in roots and leaves/shoots at different stages of barley development.

Organ	4-Day Seedlings	Developing Plant			
		Week 2	Week 4	Week 7	Week 11
Roots	0.2 ± 0.1	0.6 ± 0.1	2.0 ± 1.1	0.4 ± 0.2	N.D.
Shoots or Leaves	N.D.	0.9 ± 0.1	3.3 ± 0.1	1.1 ± 0.1	1.9 ± 0.5

N.D., signal not detected. The data represent the means ± S.D. of three technical replicates.

## Data Availability

Data are contained within the article.

## Supplementary Materials

The following supporting information can be downloaded at: https://www.mdpi.com/article/10.3390/plants15101523/s1. Table S1. Total spectral counts (TSC) of identified PLCPs detected by tandem mass spectrometry in barley seeds and 7-week-old plants, and their relative proportions (%) among all detected PLCPs. Table S2. The relative abundance of PLCPs (in %) in developing and mature (dry) barley seeds.

## References

[1] Rawlings N.D., Bateman A. (2021). How to use the MEROPS database and website to help understand peptidase specificity. Protein Sci..

[2] Grudkowska M., Zagdanska B. (2004). Multifunctional role of plant cysteine proteinases. Acta Biochim. Pol..

[3] Liu H.J., Hu M.H., Wang Q., Cheng L., Zhang Z.B. (2018). Role of papain-like cysteine proteases in plant development. Front. Plant Sci..

[4] Diaz-Mendoza M., Diaz I., Martinez M. (2019). Insights on the proteases involved in barley and wheat grain germination. Int. J. Mol. Sci..

[5] Huang J., van der Hoorn R.A.L. (2025). RD21-like proteases: Key effector hubs in plant-pathogen interactions. J. Exp. Bot..

[6] Niño M.C., Kang K.K., Cho Y.G. (2020). Genome-wide transcriptional response of papain-like cysteine protease-mediated resistance against *Xanthomonas oryzae* pv. oryzae in rice. Plant Cell Rep..

[7] Kang H., Kim S.Y., Song K., Sohn E.J., Lee Y., Lee D.W., Hara-Nishimura I., Hwang I. (2012). Trafficking of vacuolar proteins: The crucial role of *Arabidopsis* vacuolar protein sorting 29 in recycling vacuolar sorting receptor. Plant Cell.

[8] Paulus J.K., Kourelis J., Ramasubramanian S., Homma F., Godson A., Hörger A.C., Hong T.N., Krahn D., Carballo L.O., Wang S.S. (2020). Extracellular proteolytic cascade in tomato activates immune protease Rcr3. Proc. Natl. Acad. Sci. USA.

[9] Frank S., Hollmann J., Mulisch M., Matros A., Carrión C.C., Mock H.P., Hensel G., Krupinska K. (2019). Barley cysteine protease PAP14 plays a role in degradation of chloroplast proteins. J. Exp. Bot..

[10] Turk B., Turk V., Turk D. (1997). Structural and functional aspects of papain-like cysteine proteinases and their protein inhibitors. Biol. Chem..

[11] Than M.E., Helm M., Simpson D.J., Lottspeich F., Huber R., Gietl C. (2004). The 2.0 Å crystal structure and substrate specificity of the KDEL-tailed cysteine endopeptidase functioning in programmed cell death of *Ricinus communis* endosperm. J. Mol. Biol..

[12] Ahmed S.U., Rojo E., Kovaleva V., Venkataraman S., Dombrowski J.E., Matsuoka K., Raikhel N.V. (2000). The plant vacuolar sorting receptor AtELP is involved in transport of NH2-terminal propeptide-containing vacuolar proteins in *Arabidopsis thaliana*. J. Cell Biol..

[13] Martinez M., Diaz I. (2008). The origin and evolution of plant cystatins and their target cysteine proteinases indicate a complex functional relationship. BMC Evol. Biol..

[14] Hu J.J., Rampitsch C., Bykova N.V. (2015). Advances in plant proteomics toward improvement of crop productivity and stress resistance. Front. Plant Sci..

[15] Gregersen P.L., Holm P.B., Krupinska K. (2008). Leaf senescence and nutrient remobilisation in barley and wheat. Plant Biol..

[16] Distelfeld A., Avni R., Fischer A.M. (2014). Senescence, nutrient remobilization, and yield in wheat and barley. J. Exp. Bot..

[17] Díaz-Mendoza M., Velasco-Arroyo B., González-Melendi P., Martínez M., Díaz I. (2014). C1A cysteine protease-cystatin interactions in leaf senescence. J. Exp. Bot..

[18] Parrott D.L., McInnerney K., Feller U., Fischer A.M. (2007). Steam-girdling of barley (*Hordeum vulgare*) leaves leads to carbohydrate accumulation and accelerated leaf senescence, facilitating transcriptomic analysis of senescence-associated genes. New Phytol..

[19] Jukanti A.K., Heidlebaugh N.M., Parrott D.L., Fischer I.A., McInnerney K., Fischer A.M. (2008). Comparative transcriptome profiling of near-isogenic barley (*Hordeum vulgare*) lines differing in the allelic state of a major grain protein content locus identifies genes with possible roles in leaf senescence and nitrogen reallocation. New Phytol..

[20] Cohen M., Hertweck K., Itkin M., Malitsky S., Dassa B., Fischer A.M., Fluhr R. (2022). Enhanced proteostasis, lipid remodeling, and nitrogen remobilization define barley flag leaf senescence. J. Exp. Bot..

[21] Hollmann J., Gregersen P.L., Krupinska K. (2014). Identification of predominant genes involved in regulation and execution of senescence-associated nitrogen remobilization in flag leaves of field grown barley. J. Exp. Bot..

[22] Martinez M., Cambra I., Carrillo L., Diaz-Mendoza M., Diaz I. (2009). Characterization of the entire cystatin gene family in barley and their target cathepsin L-like cysteine-proteases, partners in the hordein mobilization during seed germination. Plant Physiol..

[23] Liu X.T., Mo L.J., Guo X.R., Zhang Q., Li H., Liu D., Lu H. (2021). How cysteine protease gene PtCP5 affects seed germination by mobilizing storage proteins in *Populus trichocarpa*. Int. J. Mol. Sci..

[24] Zhang N.Y., Jones B.L. (1995). Characterization of germinated barley endoproteolytic enzymes by two-dimensional gel electrophoresis. J. Cereal Sci..

[25] Wrobel R., Jones B.L. (1992). Appearance of endoproteolytic enzymes during the germination of barley. Plant Physiol..

[26] Poulle M., Jones B.L. (1988). A proteinase from germinating barley: I. Purification and some physical properties of a 30 kD cysteine endoproteinase from green malt. Plant Physiol..

[27] Koehler S.M., Ho T.H.D. (1990). Hormonal regulation, processing, and secretion of cysteine proteinases in barley aleurone layers. Plant Cell.

[28] Mikkonen A., Porali I., Cercos M., Ho T.-h.D. (1996). A major cysteine proteinase, EPB, in germinating barley seeds: Structure of two intronless genes and regulation of expression. Plant Mol. Biol..

[29] Kocks C., Maehr R., Overkleeft H.S., Wang E.W., Iyer L.K., Lennon-Duménil A.M., Ploegh H.L., Kessler B.M. (2003). Functional proteomics of the active cysteine protease content in drosophila S2 cells. Mol. Cell. Proteom..

[30] Fonovic M., Bogyo M. (2008). Activity-based probes as a tool for functional proteomic analysis of proteases. Expert Rev. Proteom..

[31] Poret M., Chandrasekar B., van der Hoorn R.A.L., Coquet L., Jouenne T., Avice J.C. (2017). Proteomic investigations of proteases involved in cotyledon senescence: A model to explore the genotypic variability of proteolysis machinery associated with nitrogen remobilization efficiency during the leaf senescence of oilseed rape. Proteomes.

[32] Havé M., Espinasse C., Cottyn-Boitte B., Puga-Freitas R., Bagard M., Balliau T., Zivy M., Ganeshan S., Chibbar R.N., Castell J.F. (2025). Triticain alpha represents the major active papain-like cysteine protease in naturally senescing and ozone-treated leaves of wheat. Plant Physiol. Biochem..

[33] Hüynck J.S., Kaschani F., van der Linde K., Ziemann S., Müller A.N., Colby T., Kaiser M., Villamil J.C.M., Doehlemann G. (2019). Proteases underground: Analysis of the maize root apoplast identifies organ specific papain-like cysteine protease activity. Front. Plant Sci..

[34] Cardoza J.D., Parikh J.R., Ficarro S.B., Marto J.A. (2012). Mass spectrometry-based proteomics: Qualitative identification to activity-based protein profiling. Wiley Interdiscip. Rev. Syst. Biol. Med..

[35] Schepetkin I.A., Fischer A.M. (2025). Activity-based profiling of papain-like cysteine proteases during late-stage leaf senescence in barley. Plants.

[36] Greenbaum D., Medzihradszky K.F., Burlingame A., Bogyo M. (2000). Epoxide electrophiles as activity-dependent cysteine protease profiling and discovery tools. Chem. Biol..

[37] Cheah J.S., Yamada S. (2017). A simple elution strategy for biotinylated proteins bound to streptavidin conjugated beads using excess biotin and heat. Biochem. Biophys. Res. Commun..

[38] Bantscheff M., Schirle M., Sweetman G., Rick J., Kuster B. (2007). Quantitative mass spectrometry in proteomics: A critical review. Anal. Bioanal. Chem..

[39] Rogers J.C., Dean D., Heck G.R. (1985). Aleurain: A barley thiol protease closely related to mammalian cathepsin H. Proc. Natl. Acad. Sci. USA.

[40] Cambra I., Martinez M., Dáder B., González-Melendi P., Gandullo J., Santamaría M.E., Diaz I. (2012). A cathepsin F-like peptidase involved in barley grain protein mobilization, HvPap-1, is modulated by its own propeptide and by cystatins. J. Exp. Bot..

[41] Diaz-Mendoza M., Dominguez-Figueroa J.D., Velasco-Arroyo B., Cambra I., Gonzalez-Melendi P., Lopez-Gonzalvez A., Garcia A., Hensel G., Kumlehn J., Diaz I. (2016). HvPap-1 C1A protease and HvCPI-2 cystatin contribute to barley grain filling and germination. Plant Physiol..

[42] Bernoux M., Timmers T., Jauneau A., Briere C., de Wit P., Marco Y., Deslandes L. (2008). RD19, an *Arabidopsis* cysteine protease required for RRS1-R-mediated resistance, is relocalized to the nucleus by the *Ralstonia solanacearum* PopP2 effector. Plant Cell.

[43] Hierl G., Vothknecht U., Gietl C. (2012). Programmed cell death in *Ricinus* and *Arabidopsis*: The function of KDEL cysteine peptidases in development. Physiol. Plant..

[44] Zhou L.Z., Höwing T., Müller B., Hammes U.Z., Gietl C., Dresselhaus T. (2016). Expression analysis of KDEL-CysEPs programmed cell death markers during reproduction in Arabidopsis. Plant Reprod..

[45] Höwing T., Dann M., Müller B., Helm M., Scholz S., Schneitz K., Hammes U.Z., Gietl C. (2018). The role of KDEL-tailed cysteine endopeptidases of *Arabidopsis* (AtCEP2 and AtCEP1) in root development. PLoS ONE.

[46] Kiyosaki T., Matsumoto I., Asakura T., Funaki J., Kuroda M., Misaka T., Arai S., Abe K. (2007). Gliadain, a gibberellin-inducible cysteine proteinase occurring in germinating seeds of wheat, *Triticum aestivum* L., specifically digests gliadin and is regulated by intrinsic cystatins. FEBS J..

[47] Otegui M.S., Noh Y.S., Martínez D.E., Vila Petroff M.G., Andrew Staehelin L., Amasino R.M., Guiamet J.J. (2005). Senescence-associated vacuoles with intense proteolytic activity develop in leaves of Arabidopsis and soybean. Plant J..

[48] Funk V., Kositsup B., Zhao C.S., Beers E.P. (2002). The *Arabidopsis* xylem peptidase XCP1 is a tracheary element vacuolar protein that may be a papain ortholog. Plant Physiol..

[49] Avci U., Petzold H.E., Ismail I.O., Beers E.P., Haigler C.H. (2008). Cysteine proteases XCP1 and XCP2 aid micro-autolysis within the intact central vacuole during xylogenesis in Arabidopsis roots. Plant J..

[50] Mulisch M., Asp T., Krupinska K., Hollmann J., Holm P.B. (2013). The Tr-cp 14 cysteine protease in white clover (*Trifolium repens*) is localized to the endoplasmic reticulum and is associated with programmed cell death during development of tracheary elements. Protoplasma.

[51] Zhang D.D., Liu D., Lv X.M., Wang Y., Xun Z.L., Liu Z.X., Li F.L., Lu H. (2014). The cysteine protease CEP1, a key executor involved in tapetal programmed cell death, regulates pollen development in *Arabidopsis*. Plant Cell.

[52] Kiyosaki T., Asakura T., Matsumoto I., Tamura T., Terauchi K., Funaki J., Kuroda M., Misaka T., Abe K. (2009). Wheat cysteine proteases triticain α, β and γ exhibit mutually distinct responses to gibberellin in germinating seeds. J. Plant Physiol..

[53] van der Linde K., Hemetsberger C., Kastner C., Kaschani F., van der Hoorn R.A.L., Kumlehn J., Doehlemann G. (2012). A maize cystatin suppresses host immunity by inhibiting apoplastic cysteine proteases. Plant Cell.

[54] Sekhon R.S., Saski C., Kumar R., Flinn B.S., Luo F., Beissinger T.M., Ackerman A.J., Breitzman M.W., Bridges W.C., de Leon N. (2019). Integrated genome-scale analysis identifies novel genes and networks underlying senescence in maize. Plant Cell.

[55] Dianoux C., Havé M., Nadam P., Leitao L., Legouge M., Leymarie J., Marmagne A., Repellin A., Freitas R.P. (2026). Dynamic of nitrogen acquisition and nitrogen partitioning in two common bean genotypes, a marker of tolerance in response to terminal drought. Plant Stress.

[56] Misas-Villamil J.C., van der Hoorn R.A.L., Doehlemann G. (2016). Papain-like cysteine proteases as hubs in plant immunity. New Phytol..

[57] Li T.T., Li Y.H., Shangguan H.B., Bian J.X., Luo R.H., Tian Y., Li Z.M., Nie X.J., Cui L.C. (2023). BarleyExpDB: An integrative gene expression database for barley. BMC Plant Biol..

[58] Hart T., Komori H.K., LaMere S., Podshivalova K., Salomon D.R. (2013). Finding the active genes in deep RNA-seq gene expression studies. BMC Genom..

[59] Zhao Y.D., Li M.C., Konaté M.M., Chen L., Das B., Karlovich C., Williams P.M., Evrard Y.A., Doroshow J.H., McShane L.M. (2021). TPM, FPKM, or normalized counts? A comparative study of quantification measures for the analysis of RNA-seq data from the NCI patient-derived models repository. J. Transl. Med..

[60] Panchy N., Lehti-Shiu M., Shiu S.H. (2016). Evolution of gene duplication in plants. Plant Physiol..

[61] Prade V.M., Gundlach H., Twardziok S., Chapman B., Tan C., Langridge P., Schulman A.H., Stein N., Waugh R., Zhang G.P. (2018). The pseudogenes of barley. Plant J..

[62] Hu G.S., Evans C.P., Satterfield K., Ellberg S., Marshall J.M., Schroeder K.L., Obert D.E. (2024). Registration of ‘GemCraft’ spring malting barley cultivar. J. Plant Regist..

[63] Schepetkin I.A., Fischer A.M. (2024). Cathepsin B- and L-like protease activities are induced during developmental barley leaf senescence. Plants.

[64] Zadoks J.C., Chang T.T., Konzak C.F. (1974). A decimal code for the growth stages of cereals. Weed Res..

[65] Leprince O., Pellizzaro A., Berriri S., Buitink J. (2017). Late seed maturation: Drying without dying. J. Exp. Bot..

[66] Wojtkiewicz M., Berg Luecke L., Kelly M.I., Gundry R.L. (2021). Facile preparation of peptides for mass spectrometry analysis in bottom-up proteomics workflows. Curr. Protoc..

[67] Nesvizhskii A.I., Keller A., Kolker E., Aebersold R. (2003). A statistical model for identifying proteins by tandem mass spectrometry. Anal. Chem..

[68] Martinez M., Gómez-Cabellos S., Giménez M.J., Barro F., Diaz I., Diaz-Mendoza M. (2019). Plant proteases: From key enzymes in germination to allies for fighting human gluten-related disorders. Front. Plant Sci..

