# Supplementary material for: Activity-Based Profiling of Papain-like Cysteine Proteases in Different Plant Organs During Barley Development
Source: Plants (Basel). 2026 May 16;15(10):1523. doi: 10.3390/plants15101523 (PMC13210622; doi:10.3390/plants15101523)
Supplement: Supplementary file 1 [file plants-15-01523-s001.zip › plants-4282591-supplementary.pdf]

Supplementary Materials
for

Activity-Based Profiling of Papain-Like Cysteine Proteases in Different Plant Organs During Barley Development

Igor A. Schepetkin and Andreas M. Fischer
Department of Plant Sciences and Plant Pathology, Montana State University, Bozeman, MT 59717, USA

Table S1. Total spectral counts (TSC) of identified PLCPs detected by tandem mass spectrometry in barley seeds and 7-week-old plants, and their relative proportions (%) among all detected PLCPs.

PLCP Name	Roots	Leaves	Stems	Seeds	Roots	Leaves	Stems	Seeds
	TSC				Fraction of PLCPs (%)			
HvPap-1	16	39	14	18	4.2	8.5	2.4	2.6
HvPap-4	7	9	58	11	1.9	2	10.1	1.6
HvPap-5	8	9	62	7	2.1	2	10.8	1
HvPap-6	118	168	168	114	31.2	36.7	29.4	16.7
HvPap-7	49	18	66	35	13	3.9	11.6	5.1
HvPap-8	5	3	4	5	1.3	0.7	0.7	0.7
HvPap-9	0	0	0	8	0	0	0	1.2
HvPap-10	0	0	0	18	0	0	0	2.6
HvPap-12	33	31	47	20	8.7	6.8	8.2	2.9
HvPap-13	4	13	12	13	1.1	2.9	2.1	1.9
HvPap-14	6	23	38	317	1.6	5.0	6.6	46.5
HvPap-16	0	5	0	0	0	1.1	0	0
HvPap-17	1	5	1	2	0.3	1.1	0.2	0.3
HvPap-19	63	71	50	54	16.7	15.5	8.7	7.9
HvPap-20	22	31	22	30	5.8	6.8	3.8	4.4
HvPap-27	5	0	0	0	1.3	0	0	0
HvPap-29	16	0	0	0	4.2	0	0	0
HvPap-30	25	32	31	29	6.6	7	5.4	4.2
HvPap-42	0	0	0	3	0	0	0	0.4
Total	378	457	573	684	100	100	100	100

Total spectral counts (TSC) from one representative technical replicate are shown. Percentages indicate each PLCP's contribution to the total TSC of all DCG-04-labeled PLCPs.

Supplementary Table S2. The relative abundance of PLCPs (in %) in developing and mature (dry) barley seeds.

Subfamily and PLCP Name	Developing Seeds	Mature Seeds
Total F-like/RD19 (HvPap-1)	2.7 ± 0.3	7.1 ± 3.5
HvPap-6	17.4 ± 0.8	23.1 ± 2.3
HvPap-7	4.7 ± 0.4	0.9 ± 0.4
Total L-like D	22.1 ± 0.9	24.0 ± 4.2
Total L-like A (HvPap-17)	0.4 ± 0.1	0.4 ± 0.3
HvPap-4	1.7 ± 0.1	2.8 ± 1.4
HvPap-5	1.3 ± 0.3	1.7 ± 0.5
Total L-Like C	3.0 ± 0.3	4.5 ± 1.5
Total XBCP3 (HvPap-8)	0.8 ± 0.2	0.4 ± 0.3
HvPap-9	1.2 ± 0.3	N.D.
HvPap-10	2.7 ± 0.2	16.5 ± 2.3
HvPap-42	0.8 ± 0.3	4.2 ± 1.1
HvPap-14	46.0 ± 0.5	24.6 ± 6.1
Total L-like B	50.7 ± 0.6	45.3 ± 6.6
Total H-like (HvPap-12)	2.6 ± 0.4	2.2 ± 0.8
Total L-like E (HvPap-13)	2.3 ± 0.4	3.2 ± 0.8
HvPap-19	7.8 ± 0.1	6.7 ± 1.0
HvPap-20	3.8 ± 0.5	1.5 ± 0.3
HvPap-30	3.8 ± 0.4	4.6 ± 0.8
Total B-like	15.4 ± 0.7	12.8 ± 1.3

The data represent the means ± S.D. of three technical replicates.
